# Improvement of burning mouth syndrome associated with Sjögren syndrome following treatment with nemolizumab

**DOI:** 10.1016/j.jdcr.2026.09.052

**Published:** 2026-10-03

**Authors:** Alina Galaria, Sydney Fritz, Dylan Wambold, Fahad Ahmed, Adel Haque

**Affiliations:** a Sidney Kimmel Medical College, Philadelphia, Pennsylvania; b Dermatology Partners, Philadelphia, Pennsylvania; c Drexel University College of Medicine, Philadelphia, Pennsylvania; d Department of Dermatology, University of Miami Health System, Miami, Florida; e Department of Dermatology, Perelman School of Medicine at the University of Pennsylvania, Philadelphia, Pennsylvania

**Keywords:** biologics, burning mouth syndrome, IL-31, nemolizumab, Sjögren syndrome

## Introduction

Burning mouth syndrome (BMS) is defined by chronic burning pain or dysesthesia of the oral mucosa lasting longer than 3 months, typically in the absence of objective clinical findings. The condition predominantly affects middle-aged and older women and is often associated with xerostomia, dysgeusia, and neuropathic symptoms. Although the etiology of BMS remains incompletely understood, proposed mechanisms include peripheral and central neuropathy, inflammatory signaling, and associations with systemic conditions such as Sjögren syndrome. Current treatments are largely symptomatic and include topical agents, systemic neuromodulators, and behavioral interventions, with inconsistent efficacy.

## Case report

### Initial presentation

A 52-year-old woman presented with a 3-year history of intermittent burning and soreness of the lips and oral mucosa, with fluctuating intensity. She described persistent lip dryness, heightened sensitivity, and burning discomfort without visible mucosal changes. She denied dry eyes, dysphagia, vaginal dryness, arthralgias, or other systemic symptoms.

Physical examination demonstrated normal-appearing oral mucosa without erythema, erosions, ulceration, or evidence of candidiasis. The lips appeared dry but intact. Given the chronicity of symptoms, laboratory evaluation was performed to assess for nutritional deficiencies, autoimmune disease, and thyroid dysfunction. While awaiting results, topical hydrocortisone 1% ointment and ketoconazole 2% cream were prescribed for symptomatic relief.

At follow-up, the patient reported persistent oral burning without improvement. Serologic evaluation revealed a positive antinuclear antibody titer of 1:160. Anti-Ro (Sjögren syndrome antigen A) and anti-La (Sjögren syndrome antigen B) antibodies were negative. Thyroid function tests (thyroid-stimulating hormone, free T4) and nutritional studies (iron, transferrin, total iron-binding capacity, folate, and vitamin B12) were within normal limits. Given concern for an underlying autoimmune process, a punch biopsy of the right inferior vermilion lip was performed.

Histopathologic examination revealed superficial perivascular dermatitis with an increased number of mast cells (Fig 1, *A*-*C*).

**Fig 1:**
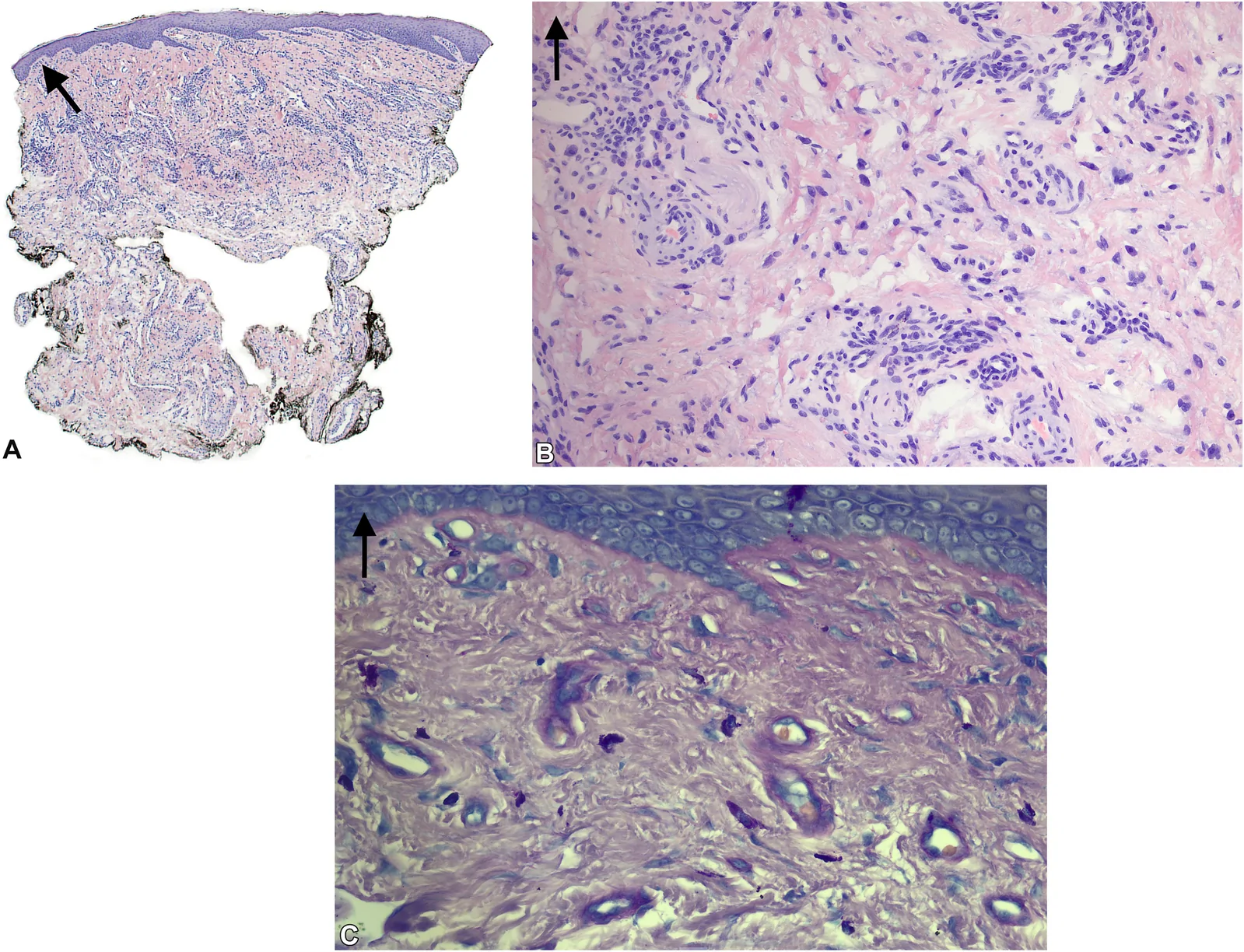
**A,** Low power (20×) H&E. **B,** High Power (200×) H&E. **C,** High Power 400× Giemsa stain highlighting an increased number of mast cells. *Arrows* point to the top of the skin.

### Management

While our patient had received a presumptive diagnosis of Sjögren syndrome from a rheumatologist previously given her symptoms of chronic dryness of the oral and ocular mucosa, she had never started any active treatment. Over subsequent months, her oral symptoms persisted despite trials of topical and systemic corticosteroids, topical tacrolimus, hydroxychloroquine, acyclovir, valacyclovir, and conservative measures, including avoidance of known irritants. The patient had not undergone treatment with pilocarpine or cevimeline.

Given refractory symptoms and significant oral discomfort, a trial of dupilumab was initiated for 4 months; however, the patient experienced minimal improvement. Dupilumab was discontinued, and nemolizumab was subsequently initiated at a dose of 60 mg administered subcutaneously, followed by 30 mg every 4 weeks thereafter.

After 8 weeks of nemolizumab therapy, the patient reported significant improvement in oral burning and pruritus. She noted a reduction in pain severity from constant moderate-to-severe discomfort to intermittent mild-to-moderate symptoms. No oral mucosal changes were observed on examination. The only adverse effect reported was fatigue.

## Discussion

BMS and Sjögren syndrome represent complex diagnostic and therapeutic challenges. BMS is a diagnosis of exclusion and may be classified as primary (idiopathic) or secondary to local or systemic conditions, including nutritional deficiencies, infections, allergic reactions, neuropathic disorders, and autoimmune disease. In this case, secondary BMS associated with suspected Sjögren syndrome was favored based on clinical symptoms, positive antinuclear antibody and supportive histopathologic findings. These findings are consistent with Sjögren syndrome, which is increasingly recognized to involve cutaneous and mucosal immune dysregulation beyond classic lymphocytic sialadenitis.^1^ Cutaneous manifestations of Sjögren syndrome may demonstrate perivascular and perieccrine lymphocytic infiltrates, interface dermatitis, and immune-mediated alterations of small nerve fibers.

Interleukin-31 is a cytokine implicated in pruritus and neuroinflammatory signaling, with elevated expression demonstrated in inflammatory and hypersensitivity disorders, most notably atopic dermatitis. Nemolizumab is a monoclonal antibody targeting the interleukin (IL) 31 receptor A, thereby interrupting downstream pruritogenic and inflammatory pathways. Although nemolizumab has primarily been studied in atopic dermatitis, its mechanism provides a biologically plausible rationale for use in pruritic conditions beyond classic eczema. In contrast to IL-4 and IL-13 blockade with dupilumab, which primarily targets type 2 inflammatory pathways, IL-31 inhibition may more directly modulate sensory nerve activation and neuroinflammatory signaling.^2^ The patient’s failure to improve with a prior 4-month dupilumab trial, followed by significant improvement with nemolizumab, may therefore suggest that IL-31–mediated neuroinflammatory pathways played a more prominent role in symptom generation.

In this context, the prominent burning and pruritic symptoms involving the oral mucosa suggest a neuroinflammatory component potentially responsive to IL-31 blockade. Although emerging literature has begun to explore nemolizumab in broader neurogenic pruritic and dysesthesia syndromes, the literature is limited for BMS specifically. Currently, there is no standardized treatment for BMS, and management has been consistently reported to be challenging.^3^, ^4^ Some studies have demonstrated partial improvement with clonazepam, capsaicin, low-level laser therapy, and cognitive behavioral therapy.^5^ This case therefore suggests that IL-31–targeted therapy may offer benefit in selected patients with refractory symptoms, particularly when pruritus is a prominent feature.

Alternative diagnoses, including oral lichen planus, candidiasis, contact dermatitis, nutritional deficiencies, and neuropathic pain disorders, were considered but deemed less likely given the normal oral examination, absence of mucosal lesions, negative infectious evaluation, and lack of histopathologic features characteristic of these conditions on biopsy. Patch testing was not performed. Beyond excluding alternative etiologies, the biopsy findings may also provide insight into underlying disease mechanisms. Mast cells play an important role in neuroimmune signaling and have been implicated in autoimmune and neuropathic pain disorders.^6^ In Sjögren syndrome, mast cell activation has been associated with local inflammation, cytokine release, and interaction with peripheral nerve endings, potentially contributing to neuropathic pain and dysesthesia.^6^, ^7^ Mast cell-derived mediators, including histamine, tryptase, and proinflammatory cytokines, may amplify sensory nerve activation and sustain chronic burning symptoms.^8^, ^9^ The increased mast cell burden observed in this patient’s biopsy therefore provides a biologically plausible link between Sjögren-associated immune dysregulation and her BMS symptoms.

## Conclusion

This case represents the first report of successful treatment of refractory BMS with nemolizumab, resulting in significant clinical improvement. The patient’s BMS occurred in the setting of suspected Sjögren syndrome, suggesting a potential link between autoimmune disease and IL-31–mediated neuropathic symptoms, as well. Further studies are needed to evaluate the safety and efficacy of IL-31 receptor inhibition in this population.

### Declaration of generative AI and AI-assisted technologies in the writing process

None.

## Conflicts of interest

None disclosed.
